# A descriptive study of patients’ and families’ experience of multifamily therapy for eating disorders in young adults

**DOI:** 10.1186/s40337-026-01730-7

**Published:** 2026-08-06

**Authors:** Berit Støre Brinchmann, Anne Kristine Sørstrøm, Lisa Marie Jacobsen, Gro Anita Ytterstad, Laurent Olivier Trichet

**Affiliations:** 1 Regional Centre for Eating Disorders, Nordland Hospital Trust, Bodø, Norway; 2 Faculty of Nursing and Health Sciences, Nord University, Postbox 1490, 8049 Bodø, Norway; 3 Nordland Hospital Trust, Bodø, Norway; 4 Faculty of Nursing and Health Sciences, Nord University, Levanger, Norway

**Keywords:** Adults, Descriptive, Eating disorder, Multifamily therapy

## Abstract

**Background:**

Eating disorders (EDs) have profound consequences not only for affected individuals but also for their families. While multifamily therapy (MFT) is well established for children and adolescents with an ED, less is known about its relevance and impact for young adults and their families. This study aimed to explore and describe experiences and outcomes of a MFT programme for young adults with a severe ED, and their family members.

**Methods:**

A descriptive multi-method design was used, combining qualitative and quantitative data collected at a regional specialist centre in Norway. Qualitative data consisted of group and individual interviews with young women with an ED, and family members who had participated in MFT between 2012 and 2022. Reflexive thematic analysis was applied. Quantitative data were collected from 47 young adult women and 99 family members before and after the MFT programme, using validated measures of ED psychopathology, mental health, quality of life, and family functioning. Descriptive and pre–post analyses were conducted.

**Results:**

Qualitative findings generated two overarching themes: “Knowledge and tools for understanding” and “Understand and be understood”. Participants described MFT as providing experiential learning, shared language, and concrete metaphors that enhanced insight into the illness and improved family communication. Quantitatively, patients reported reduced body shape concern, hostility, and trait anxiety, as well as increased satisfaction with their environment. Family members reported significantly reduced perceived burden, guilt, and need for accommodation, alongside lower levels of state anxiety following MFT.

**Conclusions:**

Findings suggest that MFT may offer meaningful benefits for both young adults with an ED and their families by strengthening understanding, communication, and emotional coping. The study extends existing evidence by highlighting the relevance of MFT beyond adolescence and underscores the importance of family-oriented interventions for young adults with a severe ED.

## Abstract (plain language summary)

EDs affect not only the person who is ill but also their whole family. Many parents and siblings feel confused, worried, and unsure how to help. Multifamily therapy (MFT) brings several families together to learn, share experiences, and support each other. In this study, we explored how young adult women with an ED and their families experienced participating in a MFT programme in Norway. We combined interviews with patients and family members, and questionnaires completed before and after the programme. Participants described MFT as helpful because it gave them new ways to understand the illness and talk about difficult topics. Creative exercises and shared discussions helped families see the ED as something separate from the person and facilitated communication and mutual understanding. The questionnaires showed that patients felt less anxious and less unhappy with their body shape, while family members felt less guilt, stress, and need to adapt their lives around the illness. Overall, the findings suggest that MFT can be a valuable form of support for young adults with an ED and their families, helping them feel less alone and better equipped to cope together.

## Background

Patients with an eating disorder (ED) often continue to live with their birth family longer than their peers [1–3]. They are suffering from a life-threatening illness carrying a significant risk of death, highest in anorexia nervosa (AN) sufferers [4], and involving dysfunctional eating patterns and body dysmorphia [5, 6] as well as, often, other psychiatric comorbidities [7], described as the nonverbal expression of underlying problems [8].

Their illness impacts family life, and relationships outside the family. Although these adverse effects are often reported, there is a paucity of evidence gathered to describe them [9]. Communication within the family may become fearful and lack hope [10]; the sufferers speak of feeling worthless as well as hopeless, of worry, depression [1] and loneliness [11].

Quality of life, not only for the sufferer but also their family members, may be reduced [12–15] not only by the illness’s negative impact on family relationships and daily living [16–19] but also by the considerable burden of caring for the sufferer. In the balance of receiving support and practicing self-care, parents’ aspiration can shift from trying to fix the ED to prioritising a relational goal of unrelenting connection with their child [20]. Family members may require professional help to cope with their emotional responses to the situation [16, 17, 21]. Help and support in managing an ED should therefore help the families of patients as well as the patient themself [22]. Complicating this, is the gap between child and adolescent mental health care, and adult mental health care [23]. Research has indicated that AN patients and their families should be supported during the transition from one to the other [24, 25], and that including the family therapeutically is helpful, challenging and intense [12, 14, 15, 25–27].

Multifamily therapy (MFT) is designed to meet this need, bringing together several families with a member suffering an ED, and bringing together various approaches to address their shared concern [28, 29]. MFT’s aim is to improve communication and interaction within the families, encouraging patients to take responsibility for their health and life, moving toward greater independence and a reduction in the symptoms of the ED [12, 29–31]. It can also help participants escape the isolation and stigma that often accompanies an ED [32, 33] leaving family members better able to speak more fully to each other [15].

Much of the research conducted into the treatment of AN and bulimia (BN) with MFT during the past twenty years has focused on children and adolescents [31–41] with little considering that of young adults [12, 27, 28, 42, 43]. What evidence we have suggests a possible improvement in the symptoms of these patients [27, 28, 34, 35, 41], gains in their weight [12, 27, 28, 34, 35, 41], improvement in both mood and self-esteem and better quality of life. MFT facilitates motivation and insight into the illness, and aids communication among members of those families affected by it. Patients and family members involved in MFT report significant treatment satisfaction, and dropout rates are low [12, 34, 35, 38, 41].

### Study setting: MFT at RESSP for eating disordered young adults

Since 2006, the Regional Centre for Eating Disorders (RESSP) at Nordland Hospital Trust in Norway has offered MFT for young adults (18–30 years) with a severe ED. There is no consensus on the definition of severe EDs [44]. In this study the diagnostic criteria for severe AN were in line with the Diagnostic and Statistical Manual of Mental Disorders, Version Five (DSM-V): body mass index (BMI) < 15; Intentional caloric restriction resulting in weight loss; intense fear of gaining weight; and body image distortion (i.e. believing themselves to be extremely fat, when they are actually normal—or even underweight). In addition, the cognitive consequences of being severely underweight impact the capacity to consent. Patients with a severe BN were included in MFT as well. The MFT programme at RESSP targeted families of young adults with a severe ED who have not yet established their own family [12, 14]. Like MFT-models used for children and adolescents, this approach is comprehensive, group based, over a twelve-month period. By actively involving families in treatment, it represents a new way of supporting young adults with a severe ED [12, 14].

The MFT programme developed by RESSP found inspiration in that offered at the Institute of Psychiatry at the Maudsley Hospital in London [45, 46]. The process of its development and inception has been described elsewhere [12, 14, 15, 30, 47]. As mentioned, the MFT programme defines young adults as being between 18 and 30-years old, and the families invited to participate must be their birth or primary family—that is, the family of young adults not yet having established a family of their own. Participating families are drawn from the catchment area, and the ED sufferer must, to participate, be under the care of a specialist therapist able to offer them follow up care. The participating family is quite broadly defined—not only parents and siblings, but others closely connected to the patient—grandparents, boy or girlfriend, and, occasionally, close friends. Family members liable to adversely affect sound group process—those suffering from severe mental illness, who are suicidal or in the grip of substance misuse/dependence—are not included. The groups formed by these families and young adults with a severe ED are led by a wide range of therapists, drawn from the in- and out-patient services within RESSP—psychologists and psychiatrists, social workers, fitness consultants, family therapists and psychiatric nurses all contribute, their levels of experience as well as backgrounds varying, leading to a diversity of approaches within the MFT group process. The MFT programme at RESSP is not so much aimed at curing the ED, seeking rather to support the patient in taking adult responsibility for it, and to support better communication and relating within the family [12, 14, 15, 30]. This differentiates it from MFT with children and adolescents (under the age of 18) suffering from a severe ED in which the family holds much more responsibility for their food intake. It is expected that every group member takes lunch together during group meetings.

The MFT programme at RESSP consists of six group meetings, each lasting two or three days, and spread over one year. This programme is eclectic, drawing on several theoretical models, principally psychodrama, systemic family therapy, collaborative therapy, mentalising-based therapy, mindfulness, multifamily therapy for psychosis and cognitive therapy. Many of the exercises used, including role-play, family mapping and sculpts and other creative work, have been developed by the RESSP team [12, 14, 15, 30].

The main topics for the group meetings are: (a) Establishing the group, (b) Communication and styles of communication, (c) Care and patterns of caring, (d) Siblings, (e) ‘Mind the gaps’ concerning transition, and (f) Summing up and ending [15]. The group divides in various ways to facilitate the exercises and interventions including role-play, family mapping and sculpts and other creative work—large groups of the patients and family members, and smaller ones (mothers, fathers, siblings, and young women with an ED). The psycho-educational and clinical groupwork sessions focus on the relevant and important themes of relationship, communication, conflict management, interactivity, motivation and feelings of guilt [14, 30].

Despite evidence supporting MFT for the families of children and adolescents with an ED [48] less is known about its significance for the families of young adults. The present study aims to address this gap by exploring the impact of MFT on young adult patients and their families, using previously collected qualitative and quantitative data, with the goal of highlighting the programme’s contribution, and illustrating the critical clinical work carried out at the treatment centre (RESSP).

## Methods

### Research design

We set up a multi-method research design involving independent but related qualitative and quantitative research approaches. The study employs a descriptive design, focusing on the experiences of young women with an ED, and of their close family members. The main body of data consists of qualitative material collected through group and individual interviews, conducted in two rounds. These interviews include participants from several MFT groups. The qualitative findings form the core of the study, while descriptive statistics are included to supplement and contextualise these insights by providing information on outcomes from MFT. The study does not seek to establish causality or causal relationships. Instead, the purpose is to capture and describe what participants—both patients and their family members—take with them from their experiences in MFT.

### The qualitative study

The qualitative study consists of two sub-studies.

#### Data collection

The first sub-study was conducted between 2015 and 2017 by BSB and consists of group interviews and individual interviews with participants in two MFT groups [15]. All participants in two MFT groups during 2015 or 2016 were invited to participate in the study, and those who wished to participate were included. Participants in the study were 12 families, six families from each of two MFT groups. Twelve young women (18 to 22 years old), eight with AN and four with BN, 12 sets of parents (mothers and fathers 43–60 years old), nine siblings (eight sisters and one brother 16–22 years old), one grandmother and two boyfriends (of the young women) participated in the two MFT groups. Some of the patients were inpatients, but most were being treated in the community. All group interviews were conducted during a fieldwork in MFT by BSB in 2015 and 2016 [15, 30, 47]. The individual interviews with parents took place in 2017(one or two years after they participated in MFT) [49]. The individual interviews with the young women took place three to four years after their participation in MFT (Table 1).

The second sub-study consists of individual qualitative interviews with eight young women with an ED, a brother and a sister, who participated in various MFT groups over a period (2012–2022), conducted by LMJ in 2024 [50]. Young women with an ED and brothers and sisters in MFT groups (during 2012–2022) were invited to participate in the study, and those who wished to participate were included. The young women with an ED were between 18 and 28 years old when they participated in MFT. The time frame between participation in MFT and research interviews in sub-study 2 varied between two and ten years. For six of the participants, the time frame was ten years, for one it was seven years, and for the rest two years (Table 1).

All the interviews were recorded on tape and transcribed.

**Table 1:** Data collection

Sub-study 1—two different MFT groups
Group interview 1 (60 min) 5 mothers, 5 fathers, MFT 1
Group interview 2 (40 min) 4 mothers, 2 fathers, one sister, one boyfriend, MFT 2
Group interview 3 (45 min) 4 young women with an ED, MFT 1
Group interview 4 (40 min) 3 young women with an ED, MFT 2
Individual interviews (60–90 min) with 6 mothers, 6 fathers, 2 young women with an ED
Sub-study 2
Individual interviews (25–60 min) with 8 young women with an ED, one brother and one sister, in various MFT groups, from 2012 to 2022

#### Data analysis

A secondary analysis (thematic analysis) of raw data from sub-study 1 [15, 30, 47] was conducted, and thematic analysis of data from sub-study 2 [50]. We used Braun and Clarke’s model of reflexive thematic analysis [51]. All interviews and group interviews were read by BSB and AS, with the research questions: What happens in MFT? How do patients with an ED experience participating in MFT? And: How do family members experience participation? We worked individually and jointly and found that key themes that recurred in the interviews concerned knowledge and understanding. BSB and AS participated in the analysis, although the main analysis was done by BSB. During the process of analysis, we moved back and forth between the six steps described by Braun and Clarke [52]: (a) Familiarising ourselves with the data, (b) Sorting the initial codes (c) Exploring themes, (d) Reviewing themes, (e) Defining and naming themes, and finally (f) Summarising and writing up. We developed two main themes: “Knowledge and tools for understanding” and “Understand and be understood.”

### The quantitative study

#### Data collection

Quantitative information regarding both patients and families has been collected at the start and end of MFT. The participants received forms during the first and last meeting of the programme and completed the forms during the following days. Among the 63 patients and 127 family members participating in the MFT programme from 2007 to 2017, 47 young adult women (75% of participants) and 99 corresponding family members (78% of participants) have completed the forms. The quantitative results have been supported by a questionnaire on common demographic or anthropometric points and pre-existing standard questionnaires on the impact of the ED on the family, on mental health related topics, on quality of life and family function (Table 2).

**Table 2:** Questionnaires

Questionnaires on the impact of the eating disorder on the family	
Accommodation and Enabling Scale (AES)	33 item self-report instrument measures caregivers’ behaviours consisting of five factors: avoidance and modifying routine, reassurance seeking, meal ritual, control of family and turning a blind eye [53] *Families only. Consistency: excellent/good. Reliability: moderate*
EDs Symptom Impact Scale (EDSIS)	A self-report instrument with 24 items to assess the impact of EDs symptoms on family members distributed in four subscales: nutrition, guilt, social isolation and dysregulated behaviour [54] *Families only. Consistency: good/good. Reliability: poor*
Questionnaires on Mental Health related topics	
Beck Depression Inventory (BDI-II)	21-item multiple-choice self-report inventory widely used measure of the presence and degree of depression in adolescents and adults. The BDI includes both cognitive and somatic symptoms of depression [55] *Patients. Consistency: excellent/excellent. Reliability: moderate* *Families. Consistency: excellent/excellent. Reliability: good*
Clinical impairment assessment due to EDs (CIA 3.0)	16-item self-report; measures the severity of psychosocial impairment due to EDs features. Domains focus on mood and self-perception, cognitive functioning, interpersonal functioning and work performance [56] *Patients only. Consistency: excellent/excellent. Reliability: moderate*
EDs Examination Questionnaire (EDE-Q 6.0)	28-item self-report questionnaire, with 6 open ended questions, focusing on the essential behavioural characteristics of EDs and 22 attitudinal questions to reflect the severity of the ED’s characteristics [57] *Patients only. Consistency: acceptable/good. Reliability: good*
Symptom Checklist 5 (SCL-5)	5-item questionnaire for general symptoms measure for anxiety and depression [58] *Families only. Consistency: good/good. Reliability: poor*
Symptom Checklist Revised (SCL-90-R)	90-item self-report screening for psychological problems and symptoms. Has three global distress indices, I. Global Severity Index (GSI) II. Positive Symptom Distress Index (PSDI) III. Positive Symptom Total (PST), and 9 subscales: somatisation, obsessive–compulsive, interpersonal sensitivity, depression, anxiety, hostility, phobic anxiety, paranoid ideation, and psychoticism [59] *Patients only. Consistency: excellent/excellent. Reliability: moderate*
State-trait Anxiety Inventory (STAI Y1, and Y2)	40-item questionnaire to diagnose anxiety and to distinguish anxiety from depressive syndromes. It has 20 items for assessing trait anxiety and 20 for state anxiety [60] *Patients. Consistency: poor/poor. Reliability: moderate* *Families. Consistency: excellent/excellent. Reliability: moderate Y1, good Y2*
Questionnaire on quality of life	
Quality of Life (WHOQOL-BREF)	Instrument with 26 items, which measures the following: physical health, psychological health, social relationships and environmental health [61–62] *Patients only. Consistency: good/acceptable. Reliability: moderate*
Questionnaire on family function	
Family functionality (Score-15)	Group self-report measures. Identifies clinically significant issues of family interaction, family functioning and change with strong psychometric properties, and the quality of family life [63] *Patients. Consistency: poor/poor. Reliability: moderate* *Families. Consistency: good/good. Reliability: moderate*

#### Implementation

The study design does not include a control group. A quasi-experimental approach is used to assess the impact of the intervention by comparing outcomes prior to and following the implementation. Such methodology and quantitative measures present power limitations, and potential implementation bias, but do offer insights and contribute to the existing knowledge base. There were variations in the method of collecting information during the study period. Depending on the years and groups, information was collected either through paper forms or through a local electronic system, called Ednor, that was set up with all the questionnaires. Forms and reply envelopes, or access codes for Ednor, were distributed at the first and last meeting. There were differences in when the participants completed and/or returned the forms, creating uncertainty about the entry date in relation to the start or end date of MFT. Registration of weight measurement in Ednor was introduced in 2013 as part of the start and completion of all measures. In general, it has been challenging to obtain objective weight measurements for multifamily patients. Even though in some cases a weight registration was made by the therapist, weight was often self-reported. Those variations in collection of information and self-reported data may lead to potential “self-selection” or “response” bias [64].

#### Statistics, quantitative analysis

The objective of the quantitative study was to confirm the profiles of the participants and support findings on the impact of MFT for our two populations. First, assess the impact of MFT intervention on Body Mass Index (BMI), ED related psychopathology, quality of life, depression, anxiety and family function in young adult women with an ED. Second, assess the impact of MFT intervention on depression, anxiety and family functionality in family members of young adult women with an ED. Descriptive statistic on our population were produced with information collected at the start of the programme, including proportions for categorical data or mean and standard deviation (SD) for continuous data. As we used a reduced sample and had no aim of generalisation, descriptive statistics do not contain inferential information such as confidence intervals. Missing data (maximum of 15% missing scores per questionnaire) were not checked as Missing At Random and were not replaced or imputed, but were excluded from tests and comparisons, and handled intrinsically by the Linear Mixed Model.

We examined potential changes during intervention by comparison of the results at the start and end of the MFT. The tests were based on parametric/non-parametric methods depending on the normality of the distributions (paired t-test, chi-square, Wilcoxon signed-rank test). Linear Mixed Model was used to adjust comparisons with demographic or health related factors. Internal consistency of the questionnaires was assessed at start and end of the MFT using Cronbach’s alpha coefficient. A value of α ≥ 0.70 was considered acceptable, α ≥ 0.80 good, and a value of α ≥ 0.90 was considered as excellent. For reliability over time, a test–retest reliability was conducted using ICC and Two-Way Mixed model. A value of ICC < 0.50 was considered as poor, ICC ≥ 0.50 moderate, ICC ≥ 0.75 good, and a value of α ≥ 0.90 was considered as excellent. Statistical analyses were performed with significance set at *p* < 0.05. Data curation, descriptive statistics, comparisons, consistency and reliability were performed using Microsoft Excel—Office 365 (Microsoft Corporation, Redmond, WA, USA), and IBM SPSS Statistics software, version 29.0 (IBM Corp., Armonk, NY, USA).

### Ethics approval and consent to participate

Our study followed the guidelines in the Declaration of Helsinki and was approved by the Regional Committee for Medical and Health Research Ethics (No. 2014/1621/No. 655808). Each participant received oral and written information about the study and signed a consent form. They were also informed that they could withdraw from the study at any time. The data were treated confidentially and anonymised.

## Results

### Findings: qualitative study

*A narrative about the ED as a sweater*

*… They were very good at visualising things, I remember very well a task like that, or they had that kind of acting almost, of what it was like. They sort of put the ED into words such as a sweater. … Now we're going to play a game where the ED is supposed to be a sweater. It sounded very silly, and the concept was a bit strange. But it was something that we, several families, have subsequently highlighted as having been a very rewarding way of looking at the ED. Yes, at least the parents understood how difficult EDs are.*

*One of the people working there was wearing a wool sweater. This was his only sweater, and it represented safety, and it kept him warm. It felt homely and it kept him both warm and feeling secure. But in some situations, this woolly jumper was perhaps a little impractical. When it rained it got wet, and when it was hot, it was too hot and began to itch. Nonetheless, there was something about it—it felt safe, as well as being the only sweater he had. But, on a hot day, it was uncomfortable to wear.*

*Now, you may have to try to replace it. Then taking it off—it was scary. It was pointed out that the ED was something so ingrained in a person, that it was safe, and felt good. While, in many situations, the ED was actually very impractical: something not quite right, some flaws like that here and there. One would have liked to have replaced it with something. And what they ended up with in conclusion was that, in a way, probably more that you didn't have to get rid of it completely. It would always be there but had several options: sort of swap it for another jacket or for a t-shirt, because an ED may always be there, because it's a part of the person. It's kind of a new thing, because I almost thought it was a monster that had taken over me. Then it is basically a part of the person themselves, that you have to learn to find new alternatives and tools that can be better suited to situations where the ED does not fit in. Yes, it became a very like, wow situation, because there were many of us who realised that it might not be as easy as just taking off this sweater, and throwing it away and then you have no other options to replace that sweater with. You haven't been to the shop, and you don't have any other jackets. Are you going to go naked? It feels so brutal, to just go from struggling with an ED, to just throwing it away, you don't know what to replace it with.*

*So, I think it was a really nice way to put it into words (young woman10, sub-study 2)*.

We developed two main themes: “Knowledge and tools for understanding” and “Understand and be understood”. The thematic areas overlap to some extent.

### Knowledge and tools for understanding

The participants said that EDs are difficult to understand. One of the young women put it this way: "*It (the disease) is insanely difficult to understand. And now I've lived with it for many years, and I still don't understand it'* (young woman 5, sub-study 2).

#### Knowledge

Several of the participants mentioned that MFT contributed to knowledge and tools for understanding. Several of the young women with an ED pointed out that the therapists were very good at communicating and had a good attitude towards the patients. They were not just seen as someone who was sick, but as people. Many mentioned that they gained new knowledge, especially about the disease and the body, but also about communication. In MFT, the therapists work directly with people's feelings, so the programme is constantly adapted based on how the participants react. MFT is not primarily about food (or the disease), but about communication and understanding in the family. You do not treat symptoms, but the underlying condition.

*"There is so much information to receive there, and it is good to gain knowledge, the right knowledge. Exchange experiences. Then you know that you are not alone, you know more about how to behave, you know the whole scenario. Because ignorance is not good, not having knowledge'* (father 4, sub-study 1).

"*Help to understand and help to communicate. I wish we had learned what we learned in MFT much earlier'* (father 6, sub-study 1).

The participants said that they cleared up misunderstandings:

*"They (the therapists) have professional knowledge that you don't have, so that's not really how it is. I remember osteoporosis and such, that you could never recover from it, and then someone says: 'Yes, you can actually recover if you eat now, but then you have to start eating now and not wait 20 years'. That there is hope, and that there is always hope of recovery'* (young woman 5, sub-study 2).

EDs are serious illnesses, and some of the information could also be frightening. One mother spoke of her two daughters. The younger sister found the information about the illness difficult and depressing. Others said that they thought knowledge was better than ignorance, but that balance was important, and that the correct use of humour could be helpful when serious information was conveyed.

"*There has been a lot of heavy material, not depressing, but very sad. But humour has helped, some have made a comment that has lightened the mood'* (parent group 2, sub-study 1).

#### Tools for understanding

Many of the participants pointed out that MFT had given them new tools that were helpful in understanding the disease, and how an ED affects the family.

*"It was these playful things we did to demonstrate what it's like to have these voices in your head. And animal figures. What kind of person are you, and how do you react when you get scared? If you back up, you leave, you stick your head in the sand. We did different exercises, we made pictures of the ED, cut pictures from magazines (collage), made figures out of plasticine, and the ED as the devil. We made pictures of the ill side, food, stress, negative words, versus the healthy side, relaxedness, nice pictures. It was nice to be in a group and exchange advice and motivation. Someone managed to put into words something you yourself had not been able to do'* (young woman 2, sub-study 1).

An "exercise" that was highlighted by several was the use of figures to illustrate the ED’s place in the family.

One brother recalled:

*"I remember this very well. We sat at a table with the family, and then we got a lot of different LEGO DUPLO people, who would describe us, here was me, here was dad, here was mum, here was little sister, here was big sister. Here was the ED then. And then we set it up how we thought it looks now, and what will it look like in 10 years? And I remember that very well. Because it was very different. Most people probably had a similar impression of how it was now, but what do you think it will look like in 10 years. I bit of that, because then we (others in the family) had thrown that skeleton LEGO DUPLO ED figure far away. But she (the ill sister) still wanted it (the disease) nearby, as a kind of 'security' perhaps'* (brother, sub-study 2).

One mother said:

"*The sibling gathering was important to our family. We used figures (together with a therapist) to illustrate how we experienced the situation. With the help of the figures.*

*(Playmo toys) they were able to show how they felt, which helped them understand each other better. This led to a conversation and improvement in the relationship, which has continued.”* (mother 1, sub-study 1).

### Understand and be understood

#### Understand

Many participants said that MFT had given them increased understanding, both about the disease, about themselves, and about each other. Many said that they had become better at putting the challenges into words and had been given '*coping strategies and a language to put into words how they felt'* (young woman 6, sub-study 2).

"*The fact that there were many people with the same thoughts and feelings. Some may have been able to find the words that they themselves were missing'* (young woman 4, sub-study 2).

"*I think it (the ED) is a disease where you learn something new every day. That's the way it is. I hear things, that you get a new understanding every time someone puts it into words, because we girls have the same disease, but we explain it in a slightly different way, which means that we get aha experiences—so I understood that—and that others might explain something, which you had but didn't know how to get it across, That’s how it is*” (young woman group 2, sub-study1).

Others described MFT as *'a light in the dark'* (young woman 8, sub-study 2).

"*I have climbed many steps in terms of knowledge and understanding and understand more of the disease—that this is the nature of the disease, it is not my daughter's delusion*" (mother 1, sub-study 1).

One young woman said: "*I think we just found a depth in each other in a way, and that we felt emotions that we hadn't felt before. And yes. I think it must be very frustrating for them. Being a relative. Not knowing what to do (*Silence*). Yes, you feel helpless then. I think that they found it difficult, and it was difficult to know that they felt it. Being the problem, that it was because of me. And that we ourselves were unable to do anything about it”* (young woman 4, sub-study 2).

Another of the young women expressed it like this: *“I thought it was very nice, because I felt that before MFT it was so difficult to put into words how I felt, it was so difficult to explain that this was not me who actively wanted to go in for being sick, it was actually deeper than that. I feel that MFT, through the lectures, the exercises we did, the conversations we had, it helped my parents understand me and the disease"* (young woman 6, sub-study 2).

#### Be understood

MFT was described as an arena for families to meet, a space where it is allowed to talk about the things that other families who do not have an ED do not understand. It creates a space where you can meet understanding, meet people who experience the same thing. The participants experienced understanding from the therapists, who had solid experience of ED and its impact on the family. Not least, they highlighted the importance of meeting others with the same disease, and families who had similar experiences as themselves.

One mother expressed it like this:

*“And it felt so good when we came to MFT, and I immediately really understood that mother and that father and that daughter. And it was mutual—because you know exactly those feelings, exactly what they say, you recognise that so well. For others, they fail to understand. Because no one understands how painful it is and how afraid you are for your daughter, and what it does to your home, and your whole family, and everyone around us. No, no one understands that” (*mother 3, sub-study 1).

Mothers and fathers' groups in particular were highlighted as important meeting places for understanding:

*"We have learned, I think we have learned from everyone. I have added and subtracted. Especially in the mothers' group, I feel. I can continue to do that; I will stop doing that. It's kind of both. I feel I have learned a lot from you”* (parent group1, sub-study1).

One of the young women with an ED described it like this:

"*You sit there and are among many. You say something, and you see that people look at you and nod, and you get a kind of confirmation and recognition for what you say, that you are understood for what you say. You get confirmation that what you say is recognisable—there is an understanding there. And others could supplement it, yes, we feel that way too. And we could also get solution-oriented answers—what other families do that is good and not good"* (young woman 1, sub-study 1).

The same participant said that MFT is so important for the family to gain an understanding, so that they can be supporters, so that the sick can get better.

"*I think that MFT was the most important thing that made me recover. MFT taught us to talk to each other”* (young woman 1, sub-study 1).

#### Summary of qualitative findings

**Fig. 1:**
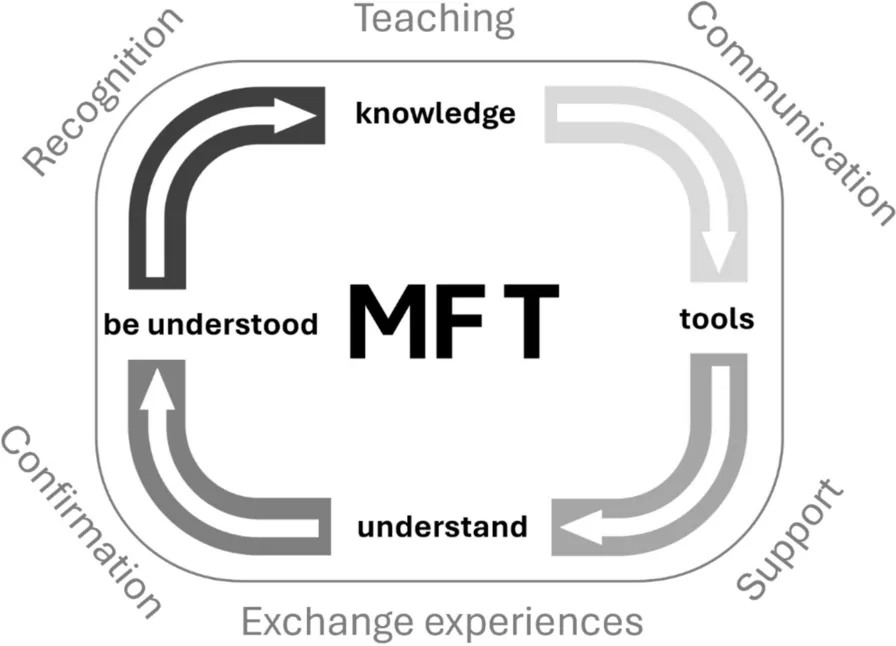
Qualitative findings

MFT is described as an ongoing process that fosters recognition, learning, and open communication, while supporting validation and the exchange of experiences. Through MFT, families gain knowledge and practical tools to better understand the nature of EDs, along with a sense of understanding and being understood (Fig. 1).

### Quantitative results

#### Changes expressed by patients during intervention

Our sample included 47 patients (Table 3), young women, 68% being between 18 and 20 years old at the start of the MFT. 76.6% were diagnosed with anorexia, with a BMI from 12 to 20, and 14.9% with bulimia, with a BMI greater than 21.

**Table 3:** Description of patients

Descriptive—patients at baseline, prior to MFT implementation		Count
Age	< 19 years old	15 (31.9%)
	19–20 years old	17 (36.2%)
	21–24 years old	8 (17.0%)
	> 24 years old	7 (14.9%)
Eating disorder diagnosis	F50.0 F50.1 Anorexia nervosa & Atypical An	36 (76.6%)
	F50.2 F50.3 Bulimia nervosa & Atypical Bu	7 (14.9%)
	F50.9 Eating disorder, unspecified	3 (6.4%)
	*Missing*	1 (2.1%)
BMI	12 to 16	11 (23.4%)
	17 to 18	8 (17.0%)
	19 to 20	5 (10.6%)
	> 21	10 (21.3%)
	*Missing*	13 (27.7%)
History of eating disorder	Number of months in past 12 months	8.8 (3.9) *
	Number of years	6.35 (4.87) *
Education level completed	Elementary school level	20 (42.6%)
	Secondary school level	20 (42.6%)
	College or University	5 (10.6%)
	*Missing*	2 (4.3%)
Main activity	Full-time and part-time work	8 (17.0%)
	On labour market measures (active support program from Labour & Welfare administration)	1 (2.1%)
	School pupil/apprentice (upper-secondary)	13 (27.7%)
	Student (university)	15 (31.9%)
	On sick leave	2 (4.3%)
	*Missing*	8 (17.0%)
Main income	From work	11 (23.4%)
	Sick pay/social security/pension	4 (8.5%)
	Supported by other	13 (27.7%)
	Scholarship/loan	12 (25.5%)
	Course allowance./Labour market measure	3 (6.4%)
	*Missing*	4 (8.5%)
Marital status	Single	36 (76.6%)
	Partner	5 (10.6%)
	*Missing*	6 (12.8%)
Living situation	With one of the parents	12 (25.5%)
	With both parents	20 (42.6%)
	Alone	6 (12.8%)
	With a partner	3 (6.4%)
	With a partner and children	1 (2.1%)
	Community	3 (6.4%)
	*Missing*	2 (4.3%)

^*^Mean (standard deviation)

When starting the MFT, the young women had an average of 6.4 years of history of ED, with an average of 8.8 months in the past 12 months.

43% had elementary school level education, 43% secondary school level and 11% high school or university level. During the intervention, approximately 60% were in school or university, while 17% had a full-time or part-time job, and 6% were on sick leave or social benefits.

No significant change in BMI was observed pre- and post-MFT in patients with anorexia nervosa or bulimia nervosa (*p* = 0.514). Changes in patient feedback before and after the intervention were confirmed by a few significant statistical differences in scores (Table 4). The differences remained confirmed when adjusted to the main descriptive variables of patients (Linear Mixed Model, using age, diagnosis, BMI, education, living condition, history of ED as additional fixed effects). The young women showed less preoccupation or dissatisfaction with body and shape after MFT (EDEQ, Body Shape Concern, score 4.65 before intervention, with time slope − 0.5 [95% CI − 0.88, − 0.12]). They showed less hostile behaviour (thoughts, feelings and actions) after the intervention (SCL90, Anger-hostility, score 58.6 before intervention, with time slope − 3.4 [95% CI − 6.7, − 0.03]). They showed less tendency to perceive stressful situations as dangerous or threatening after MFT (STAI, Trait Anxiety scale with score 57.1 before intervention and time slope − 3.9 [95% CI − 7.5, − 0.4]) and expressed more satisfaction with their environment after the intervention (QOL Environment, score 3.5 before intervention, with time slope + 0.16 [95% CI 0.01, 0.32]).

**Table 4:** Impact of intervention on patients

	Start MFT	End MFT	*p*-value
BMI	18.2 (2.1)	17.9 (2.2)	0.514
Weight	50.7 (6.4)	49.7 (6.1)	0.464
Height	166.3 (5.4)	166.8 (5.2)	0.062
CIA			
CIA Global Score	29.1 (12.4)	27.1 (14.5)	0.487
CIA Personal Impairment	12.7 (4.8)	12.2 (5.6)	0.658
CIA Social Impairment	8.6 (4.6)	7.9 (5.0)	0.529
CIA Cognitive Impairment	7.9 (4.1)	7.0 (4.6)	0.355
EDEQ			
EDEQ Global Score	3.6 (1.5)	3.4 (1.8)	0.620
EDEQ Restraint	2.9 (1.9)	3.1 (2.0)	0.731
EDEQ Eating Concern	3.0 (1.5)	2.9 (1.7)	0.747
EDEQ Body Shape Concern	4.7 (1.5)	4.1 (1.9)	0.018 *
EDEQ Weight Concern	3.8 (1.8)	3.6 (2.0)	0.747
Score15			
Family functioning Global Score	33.14(10.2)	31.4 (10.2)	0.149
Family functioning—Strength and adaptability	12.4 (4.5)	11.4 (4.5)	0.191
Family functioning—Overwhelmed by difficulties	10.7 (3.4)	9.9 (3.5)	0.153
Family functioning—Disrupted communication	10.4 (3.4)	10.1 (3.3)	0.491
SCL90			
Global Severity Index	65.2 (10.1)	63.1 (11.1)	0.389
Positive Symptom Distress Index	63.4 (8.6)	61.5 (10.0)	0.679
Positive Symptom Total	63.8 (10.5)	60.9 (12.6)	0.276
Somatization	60.5 (10.2)	59.6 (11.8)	0.708
Obsessive—compulsive	63.3 (10.9)	62.8 (12.1)	0.843
Interpersonal sensibility	64.4 (10.0)	62.1 (12.3)	0.375
Depression	64.6 (8.8)	62.2 (12.7)	0.334
Anxiety	64.0 (10.1)	62.7 (10.7)	0.579
Anger-hostility	58.6 (10.9)	55.3 (11.8)	0.048 *
Phobic anxiety	61.6 (10.0)	59.1 (10.8)	0.281
Paranoid ideation	57.7 (9.7)	56.56 (9.9)	0.593
Psychoticism	61.37 (11.7)	60.3 (11.3)	0.684
Stai			
Y1—State Anxiety Disorder	51.8 (12.4)	47.8 (16.4)	0.223
Y2—Trait Anxiety Disorder	57.1 (12.1)	53.1 (16.5)	0.043*
WHO QOL			
Physical Health	3.3 (0.6)	3.5 (0.7)	0.343
Psychological	2.4 (0.7)	2.7 (1.0)	0.228
Social relationships	3.2 (0.7)	3.3 (0.9)	0.517
Environment	3.5 (0.6)	3.7 (0.6)	0.047 *
BDI II			
BDI Total Score	26.8 (13.6)	23.4 (16.1)	0.298

^*^Significant difference between time 0 and 1, Linear Mixed Model

#### Improvements noted in family feedback

We had an average of 2 members of family per patient who replied to the questionnaires. Among 100 members, 30 did not reply to some descriptive questions (Table 5). Most members were parents, 46% mothers, 38% fathers, and 15% were siblings or “Other”. 82% of the parents were married, either living together or cohabiting. A large majority, 86%, had other children (2 or more children in the family).

**Table 5:** Description of families

Descriptive—Families at baseline, prior to MFT implementation (n = 100)		Count
Relation type with patient	Mother	33 (46%)
	Father	27 (38%)
	Sibling	5 (7%)
	Other	6 (8%)
	*Missing*	29
Civil Status	Single	10 (14%)
	Married/cohabiting	57 (82%)
	Other	3 (4%)
	*Missing*	30
Number of children	0	8 (11%)
	1	2 (3%)
	2	29 (41%)
	3 and more	31 (45%)
	*Missing*	30
Highest level of education completed	Primary level	5 (7%)
	Secondary level	25 (35%)
	University 3 years	16 (23%)
	University 4 years or more	24 (35%)
	*Missing*	30

#### Expressed improvements

The Accommodation and Enabling Scale (AES) confirmed that the families needed less adaptation to the ED after the MFT (AES, total score, score 31.5 before intervention, with time slope − 9.1 [95% CI − 12.1, − 6.2]). This perception is confirmed at the same level for mothers or fathers. The families confirmed also less impact of the ED after the intervention. They expressed feeling less guilty after the MFT and having a better experience with the nutrition situations that impact their relative with an ED (EDIS Guilt, score 10.5 before intervention, with time slope − 2.6 [95% CI − 3.6− 1.6], EDIS Nutrition, score 15.6 before intervention, with time slope − 3.1 [95% CI − 4.6, − 1.5], EDIS total score, score 33.6 before intervention, with time slope − 7.4 [95% CI, − 10.2, − 4.7]). Mothers and siblings described decreased social isolation after intervention (EDIS, Social isolation adjusted to family relation type), while fathers and others did not express any change in this feeling. All types of members showed less feelings of anxiety after MFT (STAI, State Anxiety scale with score 41.3 before intervention and time slope − 3.2 [95% CI − 6.1, − 0.3]) (Table 6).

**Table 6:** Impact of intervention on family

	Before MFT	After MFT	*p*-value
AES			
Family Adaptation—Total score	31.5 (17.2)	23.16 (13.2)	< 0.001*
BDI2			
BDI2 Total Score	12.9 (7.8)	13.1 (7.9)	0.906
EDSIS			
EDSIS Total Score	33.6 (13.9)	26.7 (12.8)	< 0.001 *
EDSIS Social isolation	6.0 (3.2)	5.6 (3.2)	0.539
EDSIS Guilt	10.5 (4.4)	8.0 (4.1)	< 0.001 *
EDSIS Nutrition	15.6 (6.6)	12.6 (6.1)	0.002 *
EDSIS Dysregulated behaviour	4.7 (3.5)	3.9 (3.3)	0.161
SCL5			
SCL5 Total Score	1.8 (1.0)	1.7 (1.0)	0.562
Score15			
Family functioning—Total Score	31.0 (8.6)	32.5 (6.7)	0.323
Family functioning—Strength and adaptability	10.9 (3.4)	11.6 (2.7)	0.280
Family functioning—Overwhelmed by difficulties	10.1 (3.5)	10.5 (2.9)	0.466
Family functioning—Disrupted communication	10.0 (3.3)	10.4 (2.6)	0.493
How do you function as a family?	3.6 (2.5)	3.6 (1.8)	0.936
How serious is it?	8.1 (2.2)	8.2 (1.4)	0.766
Treatment helpful?	2.4 (2.3)	2.1 (1.9)	0.380
Stai			
Y1—State Anxiety Disorder	41.3 (13.2)	38.2 (10.6)	0.033 *
Y2—Trait Anxiety Disorder	40.4 (10.4)	40.9 (10.4)	0.789

^*^Significant difference between time 0 and 1, Linear Mixed Model

#### Consistency and reliability of the questionnaires

A close analysis of the answers to questionnaires supports the coherency of the study. Although test–retest reliability over a one-year period was poor to moderate for AES, EDSIS, and STAI-State (Table 7), internal consistency remained high at both assessments and significant time effects were observed in the mixed models.

**Table 7:** Consistency and Reliability of questionnaires

Questionnaire	Before MFT		After MFT		Reliability before-after	
	Cronbach	Internal Consistency *	Cronbach	Internal Consistency *	ICC Two-Way Mixed model	Test–Retest Reliability **
Patients						
BDI2	0.937	Excellent	0.965	Excellent	0.666	Moderate
CIA	0.951	Excellent	0.971	Excellent	0.648	Moderate
EDEQ	0.772	Acceptable	0.835	Good	0.766	Good
SCL90	0.981	Excellent	0.985	Excellent	0.682	Moderate
Score15 Family functioning	0.395	Poor	0.387	Poor	0.674	Moderate
Stai Y1 State Anxiety Scale	0.561	Poor	0.553	Poor	0.619	Moderate
Stai Y2 Trait Anxiety Scale	0.521	Poor	0.516	Poor	0.735	Moderate
Families						
AES	0.911	Excellent	0.889	Good	0.498	Moderate
BDI2	0.925	Excellent	0.922	Excellent	0.772	Good
EDSIS	0.887	Good	0.893	Good	0.482	Poor
SCL5	0.881	Good	0.875	Good	0.398	Poor
Score15 Family functioning	0.879	Good	0.831	Good	0.642	Moderate
Stai Y1 State Anxiety Scale	0.956	Excellent	0.934	Excellent	0.592	Moderate
Stai Y2 Trait Anxiety Scale	0.935	Excellent	0.940	Excellent	0.757	Good

*Poor < 0.7, acceptable >  = 0.7, good >  = 0.8, excellent >  = 0.9

**Poor < 0.5, moderate >  = 0.5, good >  = 0.75, excellent >  = 0.9

## Discussion

The aim of this study was to explore the impact of MFT on young adult patients and their families, using previously collected qualitative and quantitative data, with the goal of highlighting the programme’s contribution, and illustrating the critical clinical work carried out at the treatment centre (RESSP) in Norway. The study examined outcomes from MFT among young adult women with a severe ED, most commonly AN, and their families. Baseline data revealed substantial illness duration, low BMI for many participants, and varying levels of educational and occupational engagement. Family participants were mainly parents with generally high educational attainment. The qualitative findings revealed two overarching themes: “Knowledge and tools for understanding” and “Understand and be understood”. Participants described MFT as providing concrete metaphors, experiential exercises, and communication strategies and coping tools that improved their insight into the nature of the illness and its impact on family dynamics. The intervention facilitated emotional expression, fostered mutual understanding, and offered a shared language for reflection. Quantitative results complemented these narratives. Patients reported reduced body shape concern, less hostility, lower trait anxiety, and greater satisfaction with their environment following MFT. Family members showed significant reduction in perceived burden and need for adaptation, less guilt, better experiences with nutrition-related situations, and lower levels of state anxiety. Together, these findings indicate that MFT provides meaningful benefits for both patients and their families by enhancing understanding, improving emotional and communicative competence, and reducing psychological distress. These results support previously published research on MFT and reinforce findings suggesting that MFT is an important therapeutic approach [26, 65]. Our study makes a distinct contribution by focusing specifically on MFT for young adults with an ED and their family members—an area that remains comparatively under-examined [26]. In addition, the quantitative results provide statistical support for family members’ and patients’ qualitative feedback, offering new perspectives to the ongoing scholarly discussion on MFT and mechanisms of change [66–69]. Such mechanisms may help explain participants’ reports of feeling less alone, learning from other families, gaining new perspectives on their own situation, and developing a shared language for discussing the illness.

Previous research has predominantly examined MFT in the treatment of adolescents with an ED, demonstrating improvements in family communication, emotional regulation, and parental self-efficacy [33, 35, 69, 70].The present findings extend this knowledge by showing that similar processes may be relevant and beneficial for young adult patients and their families. This aligns with theoretical models suggesting that family dynamics—such as expressed emotion, relational patterns, and shared meaning-making—continue to shape the course of EDs across the lifespan [10, 20, 49, 71–73]. The reported reductions in anxiety, guilt, and perceived burden among family members are consistent with studies highlighting the role of psychoeducation and structured family engagement in reducing caregiver distress [21]. Notably, the combination of qualitative and quantitative data in this study contributes to the interpretation that relational and emotional shifts within the family are key mechanisms through which MFT exerts its impact [26].

Our study is one of few studies on MFT for young adults with an ED and their families. What our study particularly contributes to is highlighting the value and utility of MFT for the population of young adults with an ED and their families, and not just for children and adolescents. The MFT programme for young adults must be developed and adapted to this target group. At the same time, MFT contributes support and knowledge to the families so that they are better equipped to take care of themselves and also support the young adult family member with an ED. In times of economic hardship, it may be that family-based services for young adults are not strengthened, but rather reduced. A direct clinical implication of our study would be to strengthen and further develop such services for young adults with an ED and their families.

### Strengths and limitations

Our study has several strengths. First, it addresses a gap in the literature by focusing on young adult patients and their families, an area that had received less attention than adolescent populations. Second, the combination of qualitative and quantitative methods provides a more comprehensive understanding of the intervention’s impact. The qualitative findings add depth and context to the quantitative results, particularly in the family dynamics. Third, the study was implemented in a real-world clinical setting that enhances the validity and relevance to clinical practice. Fourth, the family-centred approach and the inclusion of multiple family members provide valuable insights into the impact of EDs and their treatment. To a certain extent, the questionnaires analysis suggests that the instruments were internally reliable and sensitive to longitudinal change, while limited temporal stabilities reflects actual changes in the state-dependant constructs over a long follow-up period. This is particularly true for the families. However, for patients, given the large number of comparisons performed, the four significant differences should be interpreted with caution, as they were not adjusted for multiple testing and could partly reflect chance findings.

The study design also shows limitations. In the qualitative study, it was difficult to recruit informants, and it is conceivable that participants with positive experiences of MFT chose to participate in the study. Another limitation may be the large time span between participation in MFT and research interviews. Such a large time span may have affected the accuracy of what they remembered from their participation in MFT. This was particularly the case in sub-study 2, where more than half of the participants were interviewed ten years after participating in the MFT. Our study did not show a change in BMI scores, but this result may have been affected by biases in self-reported weight values. As already described, the lack of a control group prevents causal inferences, and the relatively small sample size on a specific population (young adults at RESSP in Norway) limits the generalisability of the findings. The implementation of the study implied other limitations due to potential selection bias: 25% missing patients and 22% missing family members not answering the questionnaire with no information on their specific profiles, variability in data collection methods, missing answers and to response bias (self- reported information). Lastly, the lack of long-term follow-up data limits the ability to assess the sustainability of the observed changes.

### Future research

Based on the findings of this study, different directions could be explored for future research. First, establish the causal effects of MFT on young adults with EDs and their family by conducting randomised controlled trials. Second, use longitudinal studies to examine the long-term impact of MFT on both patients and their families. This would provide insights into both the intervention’s benefits and its potential to prevent relapse in patients with an ED. Finally, future studies could examine the mechanisms of change in MFT, focusing on how specific components of the intervention contribute to the observed outcome. This would help refine the intervention and optimise its effectiveness with different populations.

## Conclusion

This descriptive multi-method study demonstrates that MFT can play a meaningful role in the treatment of young adults with a severe ED, and in supporting their families. By fostering shared understanding, experiential learning, and improved communication, MFT appears to reduce psychological distress and perceived burden among family members, while also contributing to beneficial changes in patients’ emotional well-being and illness-related concerns. Although causal conclusions cannot be drawn due to the study design, the convergence of qualitative and quantitative findings contributes to the interpretation that relational and communicative processes are central mechanisms of change in young adults with an ED. The results extend the existing literature by showing that family-based approaches remain relevant beyond adolescence and may address unmet needs among families of young adults with an ED.

## Data Availability

No datasets were generated or analysed during the current study.

## Abbreviations

AN: Anorexia nervosa
BN: Bulimia nervosa
ED: Eating disorder
MFT: Multifamily Therapy
RESSP: Regional Centre for Eating Disorders
